# Time Series Data Prediction and Feature Analysis of Sports Dance Movements Based on Machine Learning

**DOI:** 10.1155/2022/5611829

**Published:** 2022-08-24

**Authors:** DongXia Zheng, Yi Yuan

**Affiliations:** College of Physical Education, Hunan University of Science and Technology, Xiangtan 411201, China

## Abstract

Sports dance is a competition project and a kind of sports, with the characteristics of being smooth, generous, leisurely, and comfortable, dance steps, smooth movements, and flowing clouds, and it can give full play to the indoor space. In the light of the new era, sports dance is also playing an increasingly important role. Through the time series data and feature analysis of dance sports movements through machine learning, the internal information is mined to find the trends and laws. Machine learning in the era of big data is widely used in research as the main tool for data analysis and mining. The key difficulty of data mining has always been time series data. Machine learning refers to a method of using the resulting data in a computer to derive a certain model and then using this model to make predictions. The core is “using algorithms to parse data, learn from it, and then make decisions or predictions about new data.”

## 1. Introduction

This paper is based on how machine learning analyzes the time series data prediction and characteristics of dance sport movements. The process of machine learning is similar to the learning process of human beings, such as people learning mathematical theoretical models to establish logical thinking skills, analyzing and predicting things [1]. For example, chatbots–chatbots is one of the earliest forms of learning that allows humans and machines to communicate and dialogue, from which to fill the communication gap between humans and technology. For example, machines can act according to human demands or requirements [2]. The earliest is to write scripts, put the script in the machine to compile and run so that these machines have a chat function, and the script code run by the machine will make the machine recognize and let the machine according to what keywords to take what action. But there is another member of the AI family, the acceptance of machines and the use of language recognition (NLP) [3]. Let us take the interactivity and productivity generated by chatting with machines to the next level [4]. The new generation of chatbots can more effectively handle the needs of users and move forward like human-to-human communication [5]. Machine learning's algorithms are used in a wide variety of digital assistants, and this technology can be applied to the new B2C and C2C to find ways to update the traditional way of chatting with robots [6]. Communicating with robots is one of the most widely used machine learning applications in the commercial world [7]. Some AI assistant scripting languages can analyze when relevant questions need to be asked and when to ask questions and demands from humans identification classification [8]; multimedia live platform chatbots can satisfy users' use, search, and pass good music to friends and family, and at the same time, they can also enjoy the relevant music recommended by AI robots according to their personal preferences for us to enjoy [9]; during the rush hour of traffic jam, we need to take a taxi. Then you can take a taxi online through the relevant software, use the relevant software or other platforms or related request services, and receive the basic information of the driver and related vehicles that come to pick us up, such as the license plate number, color, and model of the vehicle [10]. Machine learning is also used in organizational structures, and sophisticated learning and neural networks help them analyze images [11]. Machine learning-related technologies like this which have a high breadth of applications in social media sites want to put signs on photos of other media sites, as well as road cameras and store monitoring [12]. Groups that maintain safety such as Sky Eye conduct real-time monitoring, detection, and identification of criminal behavior; later, driverless cars are driven on smooth and wide roads. Retail investors also have many applications in various aspects such as the classification of images and the recognition and analysis of images. For example, install cameras in warehouses, connect the cameras to the computer, use the computer's visual system and the self-learning system to scan the relevant items on the current shelves, and identify and determine whether there are items that are misplaced, random, or out of stock; you can also use the scanner to scan the goods taken out of the shopping cart using image recognition technology to make the goods be identified one by one. This reduces the loss of sales formed in an unintentional state; it can also be used to use image recognition in the computer through cameras, surveillance, sky eyes, and other devices to analyze whether there is suspicious activity or illegal and criminal behavior (such as smoking on high-speed rail or carrying unauthorized dangerous goods or equipment) [13]. Although most of the machine learning is highly specialized for a certain need, most merchants still try the highly specialized technology of machine learning to shorten the business process, making it easy and fast to optimize the process of collaborative processing of daily business, especially financial transactions and software development such as banks, securities companies, and other related financial transactions [14]. From the early days to the present, the most widely used applications are in the financial organization, IM, and companies' business processes, as well as software development and testing. Most departments, such as VCs and operations, are using machine learning techniques to improve the efficiency of their own departments, thereby creating more value for the company [15]. Because human energy is always limited, machine learning techniques can be used to reduce work cycles, reduce errors, and speed up work efficiency. In machine learning, we can give it a time to form a cycle according to the time we set, automatically troubleshoot errors, detect problems, and give problems to the relevant technicians in a cycle, so that we can reduce the effort on it, thereby reducing unexpected problems and interference caused by unplanned work [16]. In addition, in software testing, machine learning techniques can be added to black and white box testing and automated testing, which greatly improves the speed of software testing so that software development is faster and cheaper. Sometimes we need to extract structured critical data in documents that cannot be extracted directly, because not all data is structured and stored in unstructured and semistructured formats; that is, we need to apply NLP's machine learning technology to help us extract key structured data in related documents [17]. Experts say applying machine learning-related techniques to understand documents is a great opportunity for all aspects of life. Companies can do this, from tax returns to invoices to statutory contracts, all of which can improve efficiency and accuracy and free the work force [18] from positions that are seen as day-to-day work. Most of the smartphone's capabilities also come from machine learning. For example, voice assistants, from setting alarm clocks to finding language assistants in restaurants to decoding facial recognition phones, Apple's Siri, Xiaomi's Little Love, and Google Assistant, establish a dance sport movement time series data prediction and feature analysis based on machine learning to explore the characteristics of them for overall development.

## 2. Machine Learning for Sports Dance Movement Cognition

### 2.1. Obtain Sports Dance Movement Data

From the appearance of people, it is composed of several parts: head, neck, body, limbs, and so forth, of which the skin is on the surface of the body, and the subcutaneous tissue, muscles, and bones are below the skin. Composed of 206 bones, it can complete a variety of types, as well as uses of complex shapes; based on this physical test structure, people can complete a variety of actions; through the brain's thinking coordination, the human body in the completion of different movements reflects a strong coordination, so the study of sports dance is to make the AI system like a person have the abilities of learning, reasoning, and thinking; according to the content learned, knowledge judges what is actually going on online and predicts what will happen, hence the research on sports dance movements and intelligent control, human-computer interaction, AI, and other fields of research hotspots [19]. The capture of dances in sport includes contact and no contact in equipment, electromagnetic, inertial, machine, and optical terms, which record the movements through specific man-machine instruments, and the noncontact type includes a monocular RGB-D camera or a monocular RGB camera and a depth camera.

### 2.2. Dance Sport Movement Data Files

The dance sport movement acquisition system stores the acquired data to capture the movements in a file as BVH, which is parsed out after the action capture, which is the general format of the animation characteristic file through the human body function. It is well supported on many well-known animation software programs (flash, TV Painter, Blender3D) [20]. The representation of the human body therein is the skeleton model in the picture above, which is then expressed through the structure of the tree. BVH contains data on the movement of limb joints of characters performing dance sport movements.

## 3. Machine Learning Time Series Data Prediction Algorithm Implementation

### 3.1. Wavelet Transform

The basic solution of the wavelet transform (WT) is to represent the action signal as a set of wavelets, which can obtain information about the time and frequency domain of the action signal. The two most commonly used types of wavelet transform in WT include the continuous wavelet transform (CWT) and the discrete wavelet transform (DWT) [21].

Continuous wavelet transforms are expressed as follows:

(1) $$\begin{array}{c} W_{s\;,\tau }\left(t\right)=\int_{-\infty }^{\infty }f\left(t\right)\;\psi {*}_{s\;,\tau }\left(t\right)\text{d}t, \end{array}$$

(2) $$\begin{array}{c} \psi_{s,\tau }\left(t\right)=\frac{1}{\sqrt{\left|s\right|}}\psi \left(\frac{t-\tau }{s}\right). \end{array}$$

*ψ∗*_*s* ,*τ*_(*t*) is called a base wavelet or a mother wavelet; it is called a scaling factor (or scale); *τ* is the *ψ∗*_*s* ,*τ*_(*t*) conjugate of the translation factor. When the harmony can be continuously changed, the wavelet change at this time is called the continuous wavelet transform *ψ*_*s*,*τ*_(*t*)*sτ* CWT. Due to the complexity of the calculation of the continuous wavelet transform and the high degree of redundancy, it is not suitable for practical applications. Therefore [22], the DWT is obtained by discretizing the scaling factor in the CWT. Bring it in (2) (*s*=*s*_0_^*m*^*τ*=*n*^*τ*_0_*s*_0_^*m*^^*m*, *n* ∈ *Z*) to get discrete wavelets:

(3) $$\begin{array}{c} \psi_{m,n}\left(t\right)=\frac{1}{\sqrt{\left|s_{0}^{m}\right|}}\psi \left(s_{0}^{m}t-nb_{0}\right),\;m,n\in Z. \end{array}$$

The discrete wavelet transform is

(4) $$\begin{array}{c} W_{s,\tau }\left(t\right)=\int_{-\infty }^{\infty }f\left(t\right)\psi {*}_{s,\tau }\left(t\right)\text{d}t=\frac{1}{\sqrt{\left|s_{0}^{-m}\right|}}\int_{-\infty }^{\infty }f\left(t\right)\psi *\left(s_{0}^{-m}t-nb_{0}\right). \end{array}$$

**Figure 1:**
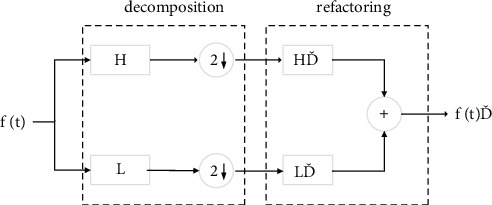
Disassembly and reconstruction of discrete wavelet transforms.

The decomposition process of DWT is equivalent to going through a high-pass filter and a low-pass filter, followed by using a binary decimation algorithm for downsampling. The DWT decomposition and reconstruction process is shown in Figure 1. H and L are decomposition filters, where H is a high-pass filter and L is a low-pass filter; after decomposition, a downsampling operation is required. H′, L′ is a reconstruction filter; likewise, H′ is a high-pass filter and L′ is a low-pass filter.

### 3.2. Static Wavelet Transform

**Figure 2:**
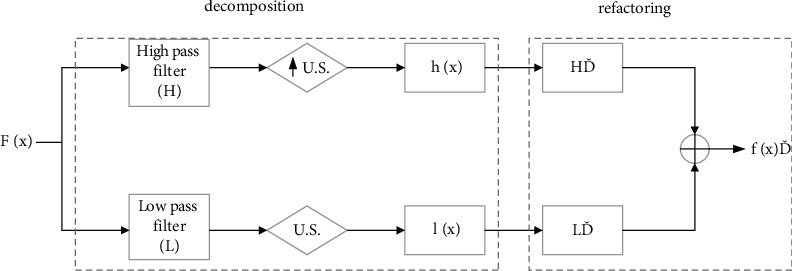
Static wavelet decomposition reconstruction.

Since the discrete wavelet decomposition uses a binary decimation algorithm to downsample the action, the wavelet coefficient will be reduced by one-half after each decomposition, so the details of the original action will be lost in each decomposition. The stationary wavelet transform (SWT) that removes the downsampling and upsamples the filter solves this problem. The SWT decomposition reconstruction process is shown in Figure 2, in which the output components of the high-pass filter and the low-pass filter are no longer downsampled, but the upper filter is upsampled to obtain a decomposition of the high-pass filter and low-pass filter in each step [23]. The detail and approximate components after each decomposition of the static wavelet are the same as the length of the original action signal, which ensures that the characteristics of the movement are preserved to the greatest extent possible and are also conducive to the analysis and study of sports dance movements.

**Figure 3:**
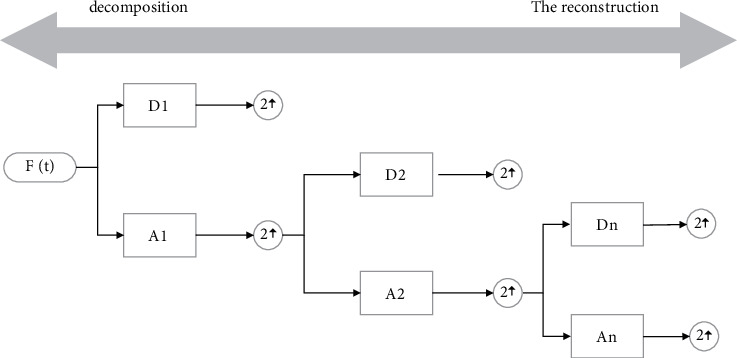
Single-step multiscale SWT decomposition process.

The single-step multiscale static wavelet decomposition process of the action signal is similar to the single-step multiscale discrete wavelet decomposition process, as shown in Figure 3.

### 3.3. ERD Models

**Figure 4:**
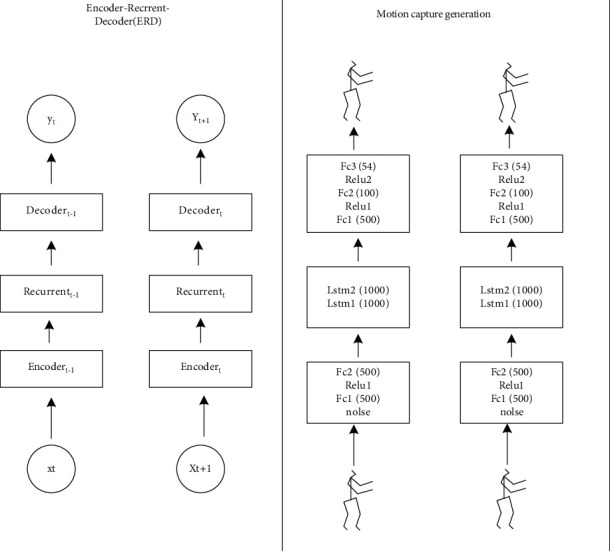
ERD model.

The ERD model was proposed by Frigidaria [24]. It is an encoder-loop unit-decoder (ERD) model used to identify and predict human posture in video and motion capture [25]. The main way it runs is to obtain a new prediction frame by continuously putting the prediction frame of the previous step into the model, so as to achieve the effect of multiframe prediction; the specific method he achieves is to encode the human action data through the fully connected network and put it into the recurrent neural network to predict the next action state according to the memory information of the previous time, and then the obtained data vector is decoded through the corresponding full connection layer, obtain the action data, and reconstruct the action through a certain reconstruction method to obtain the prediction result. Its main structure is shown in Figure 4.

### 3.4. Conversion between Dance Sport Movement Data and Trainable Data

Since the sports dance action data recorded in the dataset is in the form of quaternions, the rotation amplitude of one of the movements consists of four numbers between −1 and 1, and the continuity in the number value is not strong, so the original movement data needs to be processed to a certain extent.

That is, it proposes exponential mapping of raw data, which can effectively avoid data discontinuities and Vientiane knot locks [26]. The process is as follows:

(5) $$\begin{array}{c} \theta =r_{2},\\ r^{\prime }x=\left[\begin{array}{ccc} 0 & -r^{\prime }\left[2\right] & r^{\prime }\left[1\right]\\ 0 & 0 & -r^{\prime }\left[0\right]\\ 0 & 0 & 0 \end{array}\right],\\ r^{\prime }x=r^{\prime }x-r^{\prime }X^{T},\\ \text{rotationmat}=\left[\begin{array}{ccc} 1 & 0 & 0\\ 0 & 1 & 0\\ 0 & 0 & 1 \end{array}\right]+\sin\;\theta *r^{\prime }x+\left(1-\cos\left(\theta \right)\right)*r^{\prime }x\cdot \left(r^{\prime }x\right). \end{array}$$

To find the rotation matrix  rotationmat, where rotationmat is the original exponential mapping matrix, according to the rotation  *r*  matrix to find the rotation quaternion, the process is as follows:

(6) $$\begin{array}{c} \text{rotdiff}=R-R^{T},\\ r=\left[-\text{rotdiff}\left[1,2\right]\text{ rotdiff}\left[0,2\right]\text{ rotdiff}\left[0,1\right]\right],\\ \sin\;\theta =r_{2},\\ r0=\frac{r}{r_{2}},\\ \cos\;\theta =\frac{\text{trace}\left(R\right)-1}{2},\\ \theta =\text{arctan}\left(\frac{\sin\;\theta }{\cos\;\theta }\right),\\ \text{quaternion}=\left[\cos\left(\frac{\theta }{2}\right)\;r0\left[0\right]*\;\sin\left(\frac{\theta }{2}\right)\;r0\left[1\right]*\;\sin\left(\frac{\theta }{2}\right)\;r0\left[2\right]*\;\sin\left(\frac{\theta }{2}\right)\right] \end{array}$$

In the above equation, *R* is the rotation matrix, which eventually yields quaternion.

Due to the particularity of the heel node, if it controls the rotation mode and angle of the entire human body, we hope that the human body can have a certain stability, so the human root node is unchanged for the translation of the ground plane and for the rotation of gravity perpendicular to the ground (assuming that the ground surface is horizontal).

## 4. Case Studies

### 4.1. Comparative Experiments

The experiment selected 30 personnel for dance steps, outer dance steps, preparatory outside dance steps, reflexive movements, reflexive action positions, lifting, swinging, and other 7 kinds of sports dance movements in the same time and place to complete the experiment; the experiment does not constrain the behavior habits of the testers; participating students who take the test only need to complete the experiment in their own way. In this comparative experiment, the first adopts the traditional human movement pattern information collection method, and the second adopts the motion pattern recognition method of the accelerator and the sensor. There is a sports dance movement A, a dance step T, an outer dance step N, a preparatory outer dance step W, and a reflexive movement A. The experimental results of the reflexive action position of F lifting and descending to H and swinging to M are shown in Tables 1 and 2.

According to Tables 1 and 2, we can conclude that the difference between the dance steps and several other movements is very high, so the recognition is 100%; the difference between the reflex and reflexive action positions is very small, so its recognition is very low. Specific parameters are identified as shown in Table 3.

**Figure 5:**
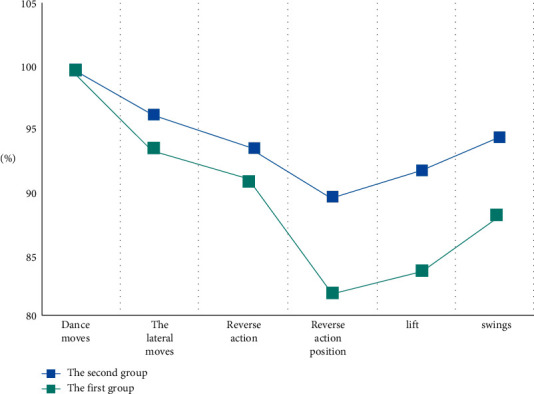
Statistical chart of recognition results.

As can be seen from Figure 5, traditional recognition technology can identify a variety of action patterns, and its recognition accuracy is balanced at 90.1%. The recognition of reflexive action and reflexive action position is low. When integrated with the accelerometer, its recognition accuracy can be balanced to 94.3%. Compared with the previous recognition accuracy, its recognition accuracy is balanced upward by 4.2 percentage points, and the result is better.

40 students were selected to test the standard degree of sports dance movements, and they were divided into two groups; that is, the regular group completed the experiment according to their own behavior, and the training group conducted sports dance movement analysis and teaching. The two sets of results are then compared and analyzed. Because the height, weight, and age of the students participating in the test are basically the same, the *P* value is greater than 0.06, so the elements related to the body will not affect the experimental results. Their situation is shown in Table 4.

**Figure 6:**
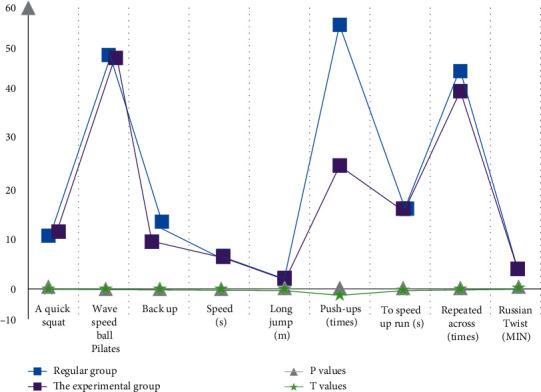
Evaluation results of preteaching SPI training indicators.

From Table 5 and Figure 6, it can be seen that the indicators of the conventional group and the training group are basically the same, and after the *t*-test is carried out on both groups, *P* is above 0.05, which depends on the initial situation of the two groups.

**Figure 7:**
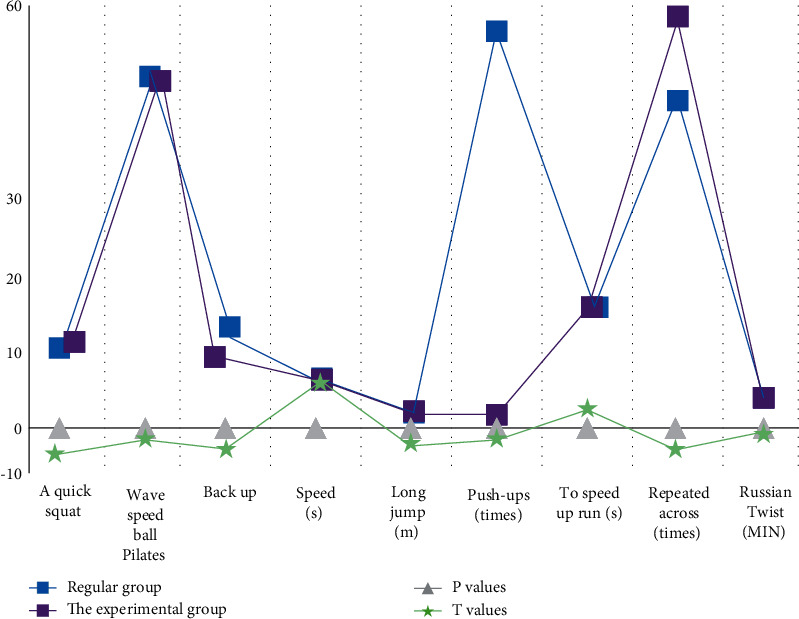
Statistics on the evaluation results of posttraining sports dance training indicators.

From Table 6 and Figure 7, it can be seen that, under the same initial conditions, the sports dance indicators of the trained students can be improved.

**Figure 8:**
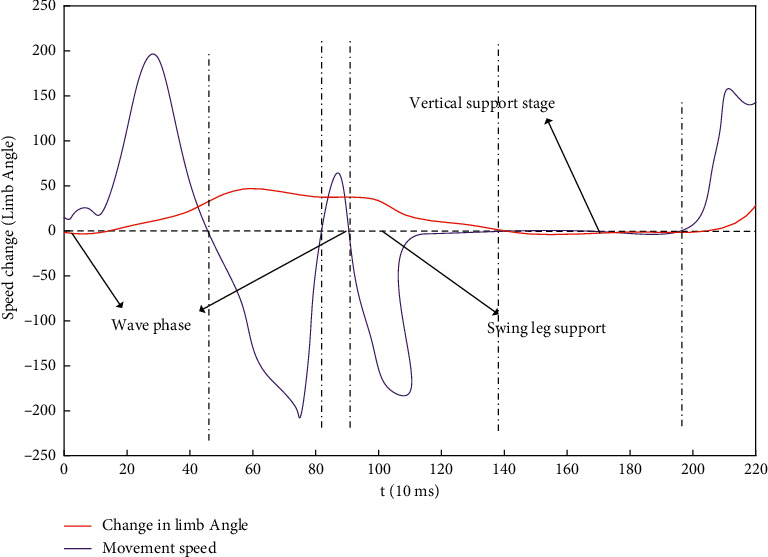
Movement angle/movement speed change curve during the dance sport movement process.

Throughout the dance movements, the angle and speed of each movement change periodically. After making appropriate modifications to the movement predictions of the time series data, the comparison curve of the angle and speed of the movements during the process of performing the dance sport movements is shown in Figure 8.

### 4.2. Data Processing

The experiments set the mean and variance of the prediction accuracy as p and *p*(1 − *p*), respectively. Due to different experiments, the prediction reversibility of the algorithm is now statistically zero, so the correct random variable with a mean of *p* is *f*, and its variance decreases as *p*(1 − *p*)/N with the increase of the repeated simulation experiment coefficient N, and when N>100, it is close to the normal distribution. This makes it possible to construct a positive-tyrannous random variable:

(7) $$\begin{array}{c} \frac{f-p}{\sqrt{p\left(1-p\right)/N}}. \end{array}$$

The following equation is then passed with the confidence level  *c*  determined:

(8) $$\begin{array}{c} P\left[-z<\frac{f-p}{\sqrt{p\left(1-p\right)/N}}<z\right]=2*\left(1-c\right). \end{array}$$

*z* values can be extrapolated from a probability table. The true value *p* can be obtained using the calculation method of probability theory, with the interval boundary value  *c*  of the probability approaching  *f* .

(9) $$\begin{array}{c} p=\frac{\left(f+z^{2}/2N\pm \sqrt{f/N-f^{2}/N+z^{2}/4N^{2}}\right)}{\left(1+z^{2}/N\right)}. \end{array}$$

± gives two values, the upper and lower bounds of the confidence.

**Figure 9:**
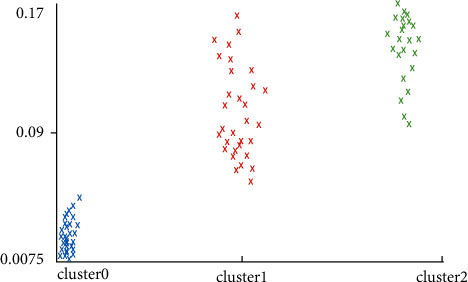
The correspondence between clustering results (horizontal axis) and action (number axis).

**Figure 10:**
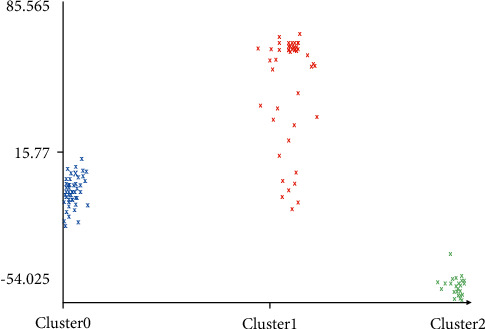
The correspondence between the clustering results and the action angle.

When collecting data, the duration of each action is relatively short, and it cannot correspond well to the data obtained individually, so, by obtaining several pieces of action cycle data, clustering is divided, and the results and data are analyzed by curve comparison to determine the action period to which the data belongs. As shown in Figures 9 and 10, the clustering results correspond to the two-dimensional three-point plot of the action.

Table 1 shows the type II prediction confusion matrix. For one class, for example, the “Dance Sport” class in Table7, set to T the correct class, and the other is the negative class F, so that the true positive (TP), true negative (TN), false positive (FP), and false negative (FN) are verified. So, the correct rate of classification of an action is

(10) $$\begin{array}{c} \text{Kappa}=\frac{TP+TN}{TP+TN+FP+FN}. \end{array}$$

The maximum value is 1, and the action is the best.

Table 8 shows the cost matrix of the three classification projections. The cost matrix represents the cost caused by prediction error and correctness, the correct cost is 0 and the cost of error is 1, so the cost of the resulting statistical error is the number of errors, as shown in Table 8.

**Figure 11:**
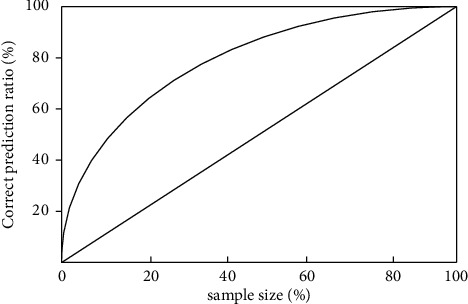
Assuming an ascending chart.

The first thing we need to consider for different movement performances is the rising chart, which represents the total number of students testing SCORP movements and the proportion of students practicing SCORP, and the vertical axis represents the correct prediction rate, as shown in Figure 11.

**Figure 12:**
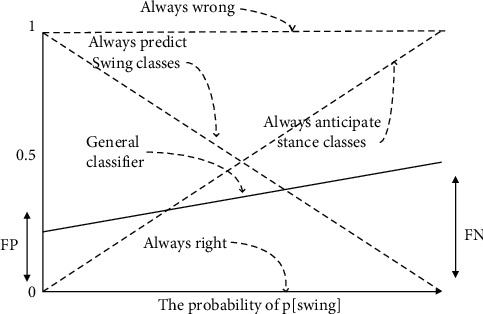
The effect of probability classification on sports movements of the sample class.

The cost curve is an action corresponding to a straight line, the purpose of which is that the action changes with the distribution of the class, as shown in Figure 12; the horizontal axis represents the probability of a certain class of samples in the training sample, and the vertical axis represents the expected error. Predictions made for only one of these types are represented by two diagonal lines, decisions are always erroneously represented by horizontal dotted lines, and horizontal lines indicate that predictions are always correct.

Weka simulation analysis will also use the working metrics in numerical forecasting to identify and propose each indicator, as shown in Tables 9 and 10.

### 4.3. Data Results Analysis

According to the experiment, the model of the article is compared with the performance of the support vector machine and the decision tree motion analysis model, and the results of three different models are observed from different aspects of accuracy and page response, and the specific experimental data are shown in Table 11.

As shown in Table 11, 8 images were taken at different distances on the same circuit board; the closest one was set as the template, and the remaining 7 groups of different images were tested to calculate the matching accuracy of various models. The method of detecting the response time of different models is to increase the number of tests and observe the average response time of different models.

**Figure 13:**
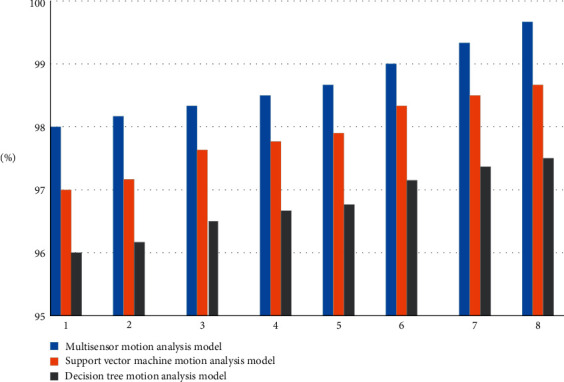
Dance sport movement model recognition accuracy.

As can be seen from Figure 13, among the 8 image samples used in our experiment, the multisensor motion analysis model has the highest accuracy, followed by the vector machine motion analysis model and the decision tree motion analysis model.

## 5. Conclusion

This paper mainly studies the use of machine learning to predict the timing data of sports dance movements and applies wavelet deformation and static wavelet variation based on the characteristics of time series data in the implementation of the algorithm. Through the implementation of the algorithm of time series data prediction, and then through the acquisition of the action data analysis model, the acquired data is transformed, and then the characteristics of the action are learned, including the encoder-loop unit-decoder (ERD) model.Then, a comparative experiment was conducted to verify this method, and the data collected were analyzed and processed to obtain the advantages of time series data prediction and feature analysis of dances in sport based on machine learning. That is, the action time series data prediction of machine learning is suitable for sports dance moves. However, due to the fact that the structure used does not have a higher level of supervision, the effect is not ideal in some aspects, although some results have been achieved here, but further research can be carried out, for example,

1. studying using seq2seq structure;
2. a combination of time and space studied using structure-run, a corresponding spatial attention model;
3. deeper use of Python and Unity3D for research.

## Figures and Tables

**Table 1:** Confusion matrix of the first set of dance sport movements.

A	T	N	W	U	F	H	M
T	190	4	2	1	2	1	0
N	1	180	7	5	7	0	0
W	0	7	81	5	6	0	1
U	0	6	7	83	4	0	0
F	2	6	3	2	87	0	0
H	5	0	0	0	3	92	0
M	5	1	0	1	0	0	93

**Table 2:** Confusion matrix of the second set of dance sport movements.

A	T	N	W	U	F	H	M
T	194	3	1	1	1	0	0
N	1	188	5	3	3	0	0
W	0	3	90	4	3	0	0
U	0	4	3	91	2	0	0
F	0	3	2	2	93	0	0
H	3	2	0	0	1	94	0
M	3	1	0	0	1	0	95

**Table 3:** Statistical table of identification results.

Mode	T (%)	N (%)	W (%)	U (%)	F (%)	H (%)	M (%)
The first group	100	95	90	81	83	87	92
The second group	100	97	94	90	91	93	94

**Table 4:** Student physical condition statistics.

	General groups	Training group	*P* value	*T* value
Height (cm)	175 2.00 ±	175 1.41 ±	1.100	−0.150
Weight (kg)	70.10 2.61 ±	70.10 2.17 ±	0.978	−0.087
Age	16.6 0.51 ±	17 0.00 ±	0.701	−2.049

**Table 5:** Evaluation results of regular dance sport training indicators.

Test the content	General groups	Training group	*P* value	*T* value
Quick squats	11.0	13.0	0.512	−0.673
Wave speed ball pilates	41.0	43.0	0.378	0.911
Pick up	9.5	14.5	0.611	−0.521
Variable speed running (s)	5.5	5.1	0.685	0.414
Long Jump (m)	1.9	2.3	0.920	0.102
Push-ups (times)	25.0	50.0	0.081	−1.880
Accelerate run (s)	18.2	17.5	0.495	−0.701
Repeatedly crossed (times)	40.0	44.0	0.262	1.168
Russian rotation (min)	4.59	4.35	0.887	−0.144

**Table 6:** Evaluation results of sports dance indicators in the training group.

Test the content	General groups	Training group	*P* value	*T* value
Quick squats	13.0	15.0	0.003	−3.578
Wave speed ball pilates	42.0	44.0	0.192	−1.372
Pick up	12.1	15.3	0.009	−3.026
Variable speed running (s)	4.6	4.2	0.000	5.062
Long Jump (m)	2.1	2.5	0.028	−2.456
Push-ups (times)	44.0	52.0	0.086	−1.864
Accelerate run (s)	18.9	17.1	0.018	2.677
Repeatedly crossed (times)	42.0	46.0	0.010	−2.973
Russian rotation (min)	4.4	4.36	0.335	0.998

**Table 7:** Category II prediction confusion matrix.

Prediction class			
		Dance sport moves (T)	Stationary (F)
Real class	Action (T)	Right (affirmatively)	Error (negation)
	Stationary (F)	Error (affirmation)	Right (negative)

**Table 8:** Three types of projected cost matrices.

Prediction class				
		a	b	c
Real class	a	0	1	1
	b	1	0	1
	c	1	1	0

**Table 9:** Numerical projection indicators.

Performance measurement	Formula
Mean square error	(*p*_1_ − *a*_1_)^2^+⋯+(*p*_*n*_ − *a*_*n*_)^2^/*n*
Root mean square error	$\sqrt{{\left(p_{1}-a_{1}\right)}^{2}+\cdots +{\left(p_{n}-a_{n}\right)}^{2}}/n$
Average absolute error	|*p*_1_ − *a*_1_|+⋯+|*p*_*n*_ − *a*_*n*_|/*n*
Relative square error	(*p*_1_ − *a*_1_)^2^+⋯+(*p*_*n*_ − *a*_*n*_)^2^/(*a*_1_ − *σ*)^2^+⋯+(*a*_*n*_ − *σ*)^2^
Relative square root error	$\sqrt{{\left(p_{1}-a_{1}\right)}^{2}+\cdots +{\left(p_{n}-a_{n}\right)}^{2}/{\left(a_{1}-\sigma \right)}^{2}+\cdots +{\left(a_{n}-\sigma \right)}^{2}}$
Relative absolute error	|*p*_1_ − *a*_1_|+⋯+|*p*_*n*_ − *a*_*n*_|/|*a*_1_ − *σ*|+⋯+|*a*_*n*_ − *σ*|
*P* is the predicted value and *a* is the true value: *σ*+1/*n*∑_*i*_*a*_*i*_	

**Table 10:** Action comparison indicators.

Index	Definition	Significance
Kappa statistic	—	Close to 100% is best
TP rate	Correct proportions	Close to 1 is best
Accuracy rate precision	*TP*/*TP*+*FP∗*100%	Close to 1 is best
Feedback rate recall	TP	Close to 1 is best
*F*-measure	2*∗TP*/2*∗TP*+*FP*+*FN*	Close to 1 is best
ROC area	—	Close to 1 is best

**Table 11:** Dance sport movement model recognition accuracy.

	1 (%)	2 (%)	3(%)	4 (%)	5 (%)	6 (%)	7 (%)	8 (%)
Multisensor motion analysis model	98	98.1	98.2	98.4	98.5	98.6	98.6	99
Support vector machine motion analysis model	97	97.2	97.4	97.6	97.7	97.8	97.9	98
Decision tree motion analysis model	96	96.2	96.3	96.4	96.4	96.5	96.7	96.8

## Data Availability

The experimental data used to support the findings of this study are available from the corresponding author upon request.
